# Cysteine Residues in *Helicobacter pylori* Adhesin HopQ Are Required for CEACAM–HopQ Interaction and Subsequent CagA Translocation

**DOI:** 10.3390/microorganisms8040465

**Published:** 2020-03-25

**Authors:** Youssef Hamway, Karin Taxauer, Kristof Moonens, Victoria Neumeyer, Wolfgang Fischer, Verena Schmitt, Bernhard B. Singer, Han Remaut, Markus Gerhard, Raquel Mejías-Luque

**Affiliations:** 1 Institute for Medical Microbiology, Immunology and Hygiene, Technical University Munich, 81675 Munich, Germany; youssef.hamway@tum.de (Y.H.); karin.taxauer@tum.de (K.T.); victoria.neumeyer@tum.de (V.N.); markus.gerhard@tum.de (M.G.); 2 Structural and Molecular Microbiology, VIB-VUB Center for Structural Biology, VIB, 1050 Brussels, Belgium; kristof.moonens@gmail.com (K.M.); han.remaut@vub.be (H.R.); 3 Department for Bioengineering Sciences, Structural Biology Brussels, Vrije Universiteit Brussel, 1050 Brussels, Belgium; 4 Med. Mikrobiology and KH-Hygiene, Max von Pettenkofer-Institute, Ludwig-Maximilians-Universität, 80336 Munich, Germany; Fischer@mvp.lmu.de; 5 Institute of Anatomy, Medical Faculty, University of Duisburg-Essen, 45147 Essen, Germany; BBSINGER@gmx.de (V.S.); verena.schmitt@uk-essen.de (B.B.S.); 6 German Center for Infection Research (DZIF), partner site Munich, 81675 Munich, Germany

**Keywords:** bacterial adhesion, CagA delivery, CEACAM1, *Helicobacter pylori*, HopQ, host-pathogen interactions, Dsb-like proteins

## Abstract

Attachment to the host gastric mucosa is a key step in *Helicobacter pylori* infection. Recently, a novel adhesin, HopQ, was shown to bind distinct host CEACAM proteins—an interaction that was found to be essential for the translocation of CagA, a key virulence factor of *H. pylori.* The HopQ–CEACAM1 co-crystal structure revealed a binding mode dependent on loops in HopQ that are clasped by disulfide bonds. In this study, we investigated the importance of these cysteine residues for CEACAM1 engagement by *H. pylori*. We observed a loss of CEACAM1 binding and CagA translocation upon disruption of the disulfide bond in loop CL1 (connecting C103 to C132 in HopQ). Deletion of the Dsb-like oxidoreductase HP0231 did not affect cell surface expression of HopQ or alter the interaction of *H. pylori* with target cells. Although HP0231 deletion was previously described to impede CagA translocation, our results indicate that this occurs through a HopQ-independent mechanism. Together, our results open up new avenues to therapeutically target the HopQ–CEACAM1 interaction and reduce the burden of pathogenic *H. pylori*.

## 1. Introduction

Infection with the gastric pathogen *Helicobacter pylori* is one of the most prevalent infections worldwide. *H. pylori* is considered a class I carcinogen, as the infection is one of the main risk factors for gastric cancer development [1]. In order to colonize and persist in the stomach, *H. pylori* needs to bind to gastric epithelial cells. A major mechanism of *H. pylori* adhesion to host cells involves the interaction between the BabA adhesin and Lewis b receptors on the gastric mucin MUC5AC [2]. However, over recent years, other important *H. pylori* adhesins have been identified, for example the outer membrane adhesin HopQ that binds to CEACAM receptors expressed on the gastric epithelium [3,4]. The binding of HopQ to CEACAM was found to be essential for *H. pylori* pathogenesis, as it is required for the type 4 secretion system (T4SS)-mediated translocation of the key virulence factor CagA into gastric epithelial cells [3,4].

Whereas BabA is a lectin that mediates binding to host carbohydrates, the HopQ-CEACAM1 structure highlighted the importance of direct protein–protein contacts for its interaction [3,5]. Interestingly, the co-complex structure revealed the presence of two cysteine-clasped loops (CL1 and CL2) that cluster together at the tip of the HopQ adhesin domain to form a platform of contact with the N-terminal domain of CEACAM1 [5,6]. Both loops are anchored by a pair of conserved cysteines. These may affect structural stability of the binding region and thereby influence attachment to CEACAM1 [5]. Other reports highlighted the importance of disulfide bond formation on the functionality of *H. pylori* virulence factors, such as the T4SS and VacA [7], and the importance of the cysteine-clasped loop in the glycan binding site of the BabA adhesin [8].

In Gram-negative bacteria, the formation of disulfide bonds occurs in the periplasm and is controlled by membrane-bound Dsb proteins [9]. The function of Dsb proteins has been extensively studied in *Escherichia coli* [9], where DsbA and DsbC play an essential role in disulfide bond formation [10,11]. In contrast, *H. pylori* possesses a relatively simple system, since it does not encode classical DsbA/DsbB or DsbC/DsbC oxidoreductases. In fact, only two extra-cytoplasmic Dsb proteins, HP0377 and HP0231, have been identified [12,13,14]. HP0377 is involved in cytochrome-c maturation and possesses disulfide isomerase activity in vitro [14]. HP0231, a major dimeric oxidoreductase of *H. pylori*, catalyzes disulfide bond formation in the periplasm [13]. HP0231 is not only important for the maintenance of redox homeostasis in *H. pylori*, but is also essential for the proper function of virulence factors possessing disulfide bonds. It has been reported that *H. pylori* lacking HP0231 are unable to translocate CagA into gastric epithelial cells [7]. In addition, VacA-mediated vacuolation is impaired upon loss of HP0231 expression and, more importantly, HP0231 is required for gastric colonization [7].

As HopQ-mediated binding to CEACAM1 involves the cysteine-clasped loops CL1 and CL2, we investigated whether the cysteines in these loops were important for HopQ stability and function. HopQ interaction with CEACAM receptors has been described as being essential for CagA translocation [3,4] and so we investigated whether the oxidoreductase HP0231, that was shown to be critical for CagA translocation, is involved in HopQ disulfide bond formation. Our results indicate that disruption of the disulfide that stabilizes CL1 abolishes HopQ-mediated CEACAM binding. However, we found that HopQ expression and adherence to host CEACAM receptors are independent of HP0231.

## 2. Materials and Methods

### 2.1. H. pylori Strains and Generation of Mutant Strains

*H. pylori* P12 [15], P12 ∆*hopQ* [4], P12 ∆*hp0231* [7], P12 ∆*hp0231* + *hp0231* [7], G27 [16], G27 ∆*hopQ* [3] and G27 ∆*hp0231* [7] were used in this study.

To generate *H. pylori* complemented with wild type HopQ or point mutants, HopQ was cloned into the pWS241 vector using Gibson Assembly (NEB), and point mutants (C103S, C132S, C238S and C362S) of the gene generated using the Quikchange system (Agilent, Santa Clara, CA, USA). The resulting plasmids were transformed into *H. pylori* G27 ∆hopQ. In brief, bacteria grown on WC Dent plates for two days were collected and resuspended in Brain Heart Infusion (BHI) medium, 2µg of plasmid were added to 200 µl of this suspension, mixed, replated on WC Dent plates, and allowed to grow for 24 h. Bacteria were then transferred to WC Dent plates containing 50μg/mL kanamycin to select for bacteria harboring the plasmid. The expression of HopQ was verified by Western blot (Figure 2a).

### 2.2. Cells

MKN28 [17] vector control, MKN28-CEACAM1-4L, AGS (ATCC, CRL-1739), CHO (ATCC, CCL-61) vector control, and CHO-CEACAM1-4L cells [3] were maintained in an incubator at 37 °C and 5% CO_2_. Cells were grown in DMEM (Gibco, Thermo Fisher Scientific, Waltham, MA, USA) containing 2 mM L-glutamine (Gibco) supplemented with 10% FBS (Gibco) and 1% penicillin/streptomycin (Gibco).

### 2.3. Infections and Western Blot for CagA Translocation

Cells were seeded at a density of 7.5 × 10^4^ in a 24-well plate, two days before infection. On the day of the infection, one well was trypsinized and harvested cells were counted.

*H. pylori* grown on WC Dent plates for two days were collected, resuspended in BHI medium, and counted based on their optical density (OD_600_), with OD_600_ = 1 = 2 × 10^8^ bacteria. The number of bacteria required for a multiplicity of infection (MOI) of 50 was determined, added to the eukaryotic cells and incubated for 6 h at 37 °C /5% CO_2_. After infection, the cells were washed 1x with PBS, lysed with Laemmli buffer + 50mM DTT, and run in an SDS-PAGE on 6% polyacrylamide gels followed by a Western blot onto nitrocellulose membranes (GE Healthcare, Chicago, IL, USA). The Western blot membrane was blocked with 5% skim milk (Carl Roth, Karlsruhe, Germany)/PBST probed with mouse anti-pTyr (1:300), rabbit anti-CagA serum (1:3000), and rabbit anti-GAPDH (1:1000) or anti-alpha tubulin (1:1000, loading control) in 5% Bovine serum albumin (Applichem, Darmstadt, Germany)/PBST. The CagA blot was reprobed with the anti-pTyr antibody to detect phosphorylated CagA.

### 2.4. Pull Down Assays

*H. pylori* grown on WC Dent plates for two days were collected and their optical density measured. The cultures were adjusted to OD_600_ = 0.5 in 1 mL of media, and 2 µg CEACAM1-Fc recombinant protein [18] was added. The mixture was left to shake for 2 h in a microaerobic incubator, followed by washing 3× with PBS. The washed sample was lysed in Laemmli buffer + 50 mM DTT, run in an SDS-PAGE followed by a Western blot and the membrane was probed for CEACAM1 (SAB3 antibody, 2.5 µg/mL) and HopQ (mouse serum, 1:3000) as a control.

### 2.5. Binding Assay (Flow Cytometry)

*H. pylori* grown on WC Dent plates for two days were collected and their optical density measured. The cultures were adjusted to OD_600_ = 1 in 1 mL of media and stained with 10 µM Carboxyfluorescein succinimidyl ester (CFSE, eBioscience, San Diego, CA, USA) for 30 min. The bacteria were washed twice with PBS, counted once more, and added to AGS or pcDNA3.1 empty vector- or CEACAM1-4L-transfected MKN28 or CHO cells at MOI 10. The mixture was incubated in a microaerobic incubator, shaking for 30 min; followed by washing four times with PBS. After washing, the samples were resuspended in 1% PFA/PBS and acquired on a Cyan ADP or CytoFLEX S flow cytometer (Beckman Coulter, Brea, CA, USA). The eukaryotic cells were gated based on forward and side scatter, and their fluorescence measured in the FITC channel. This quantified the proportion of the gated cells bound by the fluorescent bacteria. 100% indicates that each eukaryotic cell exhibited fluorescence *H. pylori* binding.

### 2.6. Immunofluorescence

1.5 × 10^5^ MKN28-CEACAM1-4L or CHO-CC1 were seeded on coverslips in a 12-well plate and grown for two days. *H. pylori* grown on WC Dent plates for two days were collected, counted and CFSE-stained as described above. The CFSE-labeled bacteria were then used to infect the cells at MOI 10 for 3 h. After infection, the cells were methanol/acetone-fixed, phalloidin-stained and mounted with DAPI-containing medium (Vector Labs, Burlingame, CA, USA). Pictures were taken using a confocal microscope SP5 (Leica, Wetzlar, Germany).

### 2.7. Thermal Denaturation Assay

Solutions of 7.5 μL of 300 × SYPRO Orange (Invitrogen, Carlsbad, CA, USA), 5 μL of 2.5 mg/mL HopQ type I adhesin domain (HopQ^AD-I^, comprising residues 22–425 of the mature protein, for production details see [5]) and 12.5 μL of a dithiothreitol (DTT) dilution series or buffer (20 mM Tris HCl pH 8.0, 150 mM NaCl and 5% (*v*/*v*) glycerol) were added to the wells of a MicroAmp Optical 96 well Reaction Plate (Applied Biosystems, Foster City, CA, USA). The plates were sealed with Microamp optical adhesive film (Applied Biosystems) and heated in a 7500 Real Time PCR System (Applied Biosystems) from 4 to 95 °C in increments of 1 °C. Fluorescence changes in the wells of the plate were monitored simultaneously and plotted against temperature. The wavelengths for maximal excitation and emission of the SYPRO orange are 470 and 570 nm, respectively.

### 2.8. CEACAM-GFP Binding Assay

*H. pylori* grown on WC Dent plates for two days were collected and their optical density measured. The cultures were adjusted to OD_600_ = 1 in 1 mL of media and stained with 5 µM Cell Proliferation Dye eFluor™ 670 (eBioscience) for 30 min. The bacteria were washed twice in PBS, and evenly divided into 8 wells of a 96 well plate, to which 3 µg of the respective CEACAM-GFP (or GFP control) [19,20,21] were added. The mixture was incubated in a microaerobic incubator, and shaken for 30 min; followed by washing 4x with PBS. After washing, the samples were fixed in 1% PFA/PBS and acquired on a CytoFLEX S flow cytometer.

### 2.9. H. pylori Outer Membrane Preparation

*H. pylori* strains were grown as described above, then 100 mg of bacteria was collected and resuspended in 300 µl of buffer (20 mM Tris, 500 mM NaCl, pH 8 + 2mM EDTA, 100 µg/mL lysozyme, 20 µg/mL DNAase and cOmplete™, Mini, EDTA-free Protease Inhibitor Cocktail (Roche, Basel, Switzerland)). This was followed by 4× freeze–thaw cycles using dry ice and a water bath to lyse the bacteria. The lysed bacteria were then centrifuged at 10,000× *g*, 4 °C for 15 min. The supernatant was collected and centrifuged at 20,000× *g*, 4ºC for 90 min. The pellet was retained and dissolved in 200 µL of the above buffer + 0.5% *N*-lauroylsarcosine and incubated for 30 min at RT, shaking. This solution was centrifuged once more at 20,000× *g*, 4 °C for 90 min. The pellet was retained and dissolved in Laemmli buffer + 50 mM DTT, run in an SDS-PAGE followed by a Western blot and the membrane was probed for HopQ as above, with HpaA as a positive control (mouse serum, 1:3000) and gGT as a negative control (mouse serum, 1:3000).

### 2.10. List of Antibodies Used

Mouse anti-pTyr (1:300, PY99, Santa Cruz Biotechnology, Dallas, TX, USA);
Rabbit anti-CagA serum (1:3000);
Rabbit anti-GAPDH (1:1000, loading control, 14C10, Cell Signaling Technology, Danvers, MA, USA);
Rabbit anti-alpha tubulin (1:1000, loading control, polyclonal, Cell Signaling Technology);
SAB3 antibody (anti-CEACAM1/3/5 from B.B. Singer), 2.5 µg/mL;
Mouse anti-HopQ serum (1:3000);
Mouse anti-HpaA serum (1:3000);
Mouse anti-gGT serum (1:3000).

## 3. Results

### 3.1. Disulfide Bridges in the HopQ Adhesin Domain are Essential for CEACAM1 Binding

**Figure 1:**
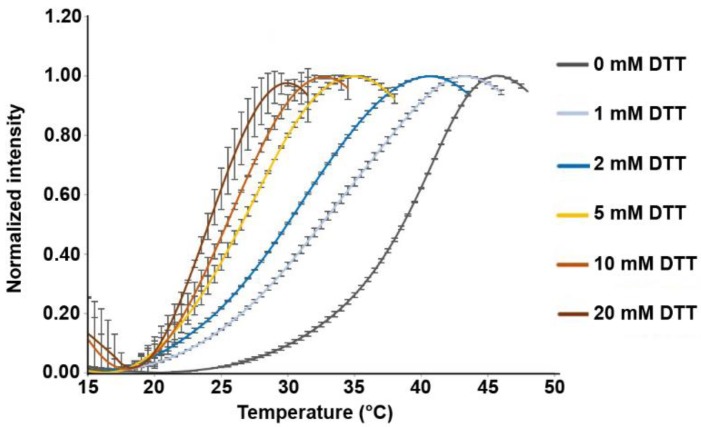
Disulfides are critical for HopQ stability. Thermal shift (Thermofluor) assay measuring the thermal unfolding of HopQ in the presence of a range of DTT concentrations. Error bars show S.D.

To assess whether disulfide bond formation is important for HopQ stability, we performed a thermal stability (Thermofluor) assay on the HopQ adhesin domain (HopQ^AD^, comprising residues 22–425 of the mature protein) in the presence of different concentrations of the reducing reagent dithiothreitol (DTT). We observed that even small amounts of DTT (1–2 mM) were able to destabilize HopQ^AD^, as evidenced by the shift of the Thermofluor peak (Figure 1). Increasing concentrations of DTT even further reduced HopQ^AD^ thermal stability until around 30 °C, where a plateau was reached in the range of 10–100 mM DTT.

**Figure 2:**
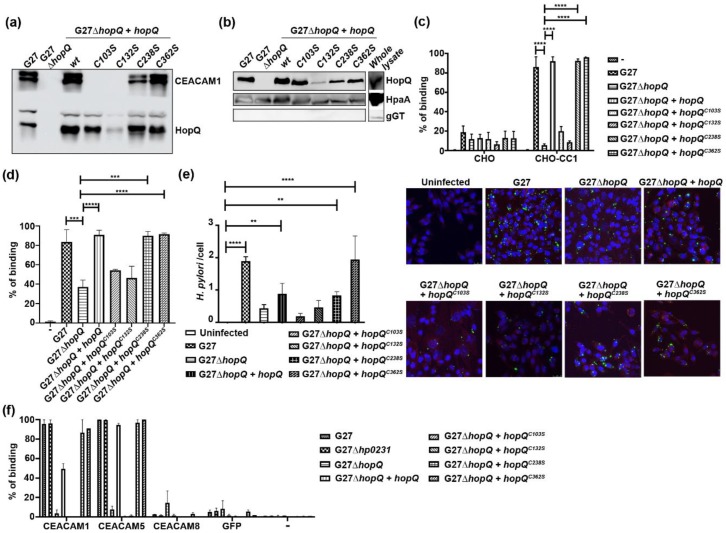
An intact CL1 disulfide in HopQ is crucial for CEACAM1 binding. (**a**) Western blot showing pull down pattern of recombinant CEACAM1 using the wild type *H. pylori* strain G27, G27 ∆*hopQ*, or G27 ∆*hopQ* strains that are complemented with either wild type or different mutant *hopQ* alleles. (**b**) HopQ protein levels detected by Western blot in outer membrane extracts of different *H. pylori* strains. HpaA and gGT were used as positive and negative controls for the membrane preparation, respectively. (**c**) and (**d**) Flow cytometry cell-binding assay. Bar graph showing the percentage of CHO cells (carrying a vector control or expressing CEACAM1-4L) (**c**) or MKN28 cells (**d**) bound by fluorescently labelled *H. pylori* from panel (**a**). ‘-’ denotes cells with no bacteria added. (**e**) Confocal microscopy and quantification of fluorescently labeled G27, G27 ∆*hopQ*, or G27 ∆*hopQ* strains complemented with either wild type or different mutant *hopQ* alleles binding to CHO-CEACAM1-4L (CC1) cells. Scale bar 25 µm. Magnification = 63×. (**f**) Flow cytometry *H. pylori* binding assay. Bar graph showing the percentage of *H. pylori* binding to CEACAM-GFP. ** *p* ≤ 0.01, *** *p* ≤ 0.001, **** *p* ≤ 0.0001.

Our results indicate that HopQ stability depends on disulfide bond formation. Therefore, loss of disulfides might impact HopQ functionality. HopQ^AD^ possesses three different cysteine-clasped loops CL1 (anchored by residues C103 and C132); CL2 (anchored by residues C238 and C270); and CL3 (anchored by residues C362 and C385), of which CL1 and CL2 were likely to influence HopQ binding activity as deduced from the HopQ-CEACAM1 co-complex structure [5]. To test this hypothesis, we generated cysteine to serine mutant strains C103S, C132S, C238S and C362S (from CL3, as a negative control), and tested binding to CEACAM1 in pull down experiments. We observed that CL1 mutants (C103S and C132S) reduced CEACAM1 binding to the levels of the ∆*hopQ* mutant (Figure 2a), while the CL2 and CL3 mutants (C238S and C362S) had no effect on the pull-down of CEACAM1 (Figure 2a). Of note, reduced surface expression of the C132S mutant was detected, which could account for the reduced binding to CEACAM1 that was observed for this strain (Figure 2b); however, this is in keeping with the overall reduced expression of HopQ by this strain (Figure 2a). In contrast, similar levels of HopQ were observed in membrane preparations of the C103S mutant compared to the wild type strain (Figure 2b). This suggests that the observed reduction in binding is based on these mutations altering the direct interaction of HopQ to CEACAM1, rather than being a result of structural changes that prevent outer membrane expression of the protein. Together, these results suggest an important role for the CL1 disulfide bridge in CEACAM1 binding.

We next performed binding assays to CHO cells stably transfected with CEACAM1-4L (CHO-CEACAM1-4L) [22]. A parental CHO cell line that does not express any CEACAM proteins was used as a negative control. In agreement with the loss of CEACAM1 interaction observed with the pulldown assay (Figure 2a), flow-cytometry-based binding assays showed that the binding of *H. pylori* cells expressing HopQ mutants C103S or C132S to CHO-CEACAM1 cells was reduced to the levels seen for the ∆*hopQ* mutant (Figure 2c). Similar results were obtained when using MKN28 cells (Figure 2d), thus confirming the importance of the CL1 disulfide on a cellular level. We also confirmed these observations using confocal microscopy (Figure 2e). Together, these results indicate that disulfide bond formation, specifically in CL1, is critical for HopQ stability and function. 

HopQ has also been shown to bind CEACAM5 [3]. To determine whether disulfide formation would also affect the binding of *H. pylori* to CEACAM5, we tested binding of the various cysteine mutants to recombinant CEACAM5-GFP. We found that loss of the CL1 disulfide bond similarly affected binding (Figure 2f).

### 3.2. H. pylori Oxidoreductase HP0231 is Not Required for HopQ Binding to Gastric Cells

**Figure 3:**
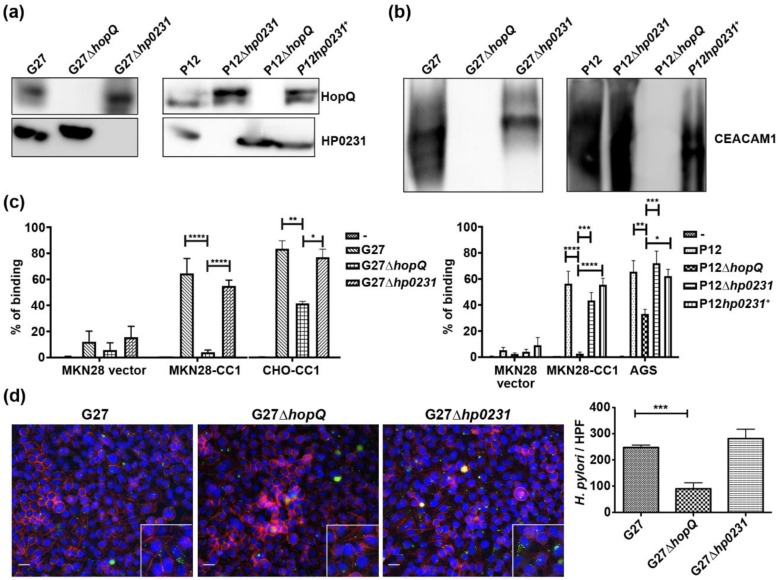
Deletion of HP0231 does not influence HopQ expression, stability or its CEACAM1 binding capacity. (**a**) Western blot of different *H. pylori* strains, probed with anti-HopQ and anti-HP0231 serum. (**b**) Pulldown of recombinant CEACAM1-Fc by several *H. pylori* strains. (**c**) Flow cytometry binding assay of same *H. pylori* strains to CEACAM1-deficient MKN28 cells, CEACAM1-4L-transfected MKN28 cells, CHO-CEACAM1-4L (CC1) cells, and AGS cells. ‘-’ denotes cells with no bacteria added. (**d**) Immunofluorescence microscopy and quantification of G27 wild type, ∆*hopQ* and ∆*hp0231 H. pylori* strains binding to MKN28-CEACAM1-4L cells. Scale bar 25 µm. HPF, high-power field. * *p* ≤ 0.05, **p ≤ 0.01, *** *p* ≤ 0.001, **** *p* ≤ 0.0001.

Knockout mutants of the *H. pylori* oxidoreductase HP0231 showed a CagA translocation defect, similar to what is seen in *hopQ* null mutants [3,7]. Since we found the CL1 disulfide to be important for HopQ functionality, we checked whether the CagA translocation defect seen in HP0231 mutants was a result of a compromised disulfide formation in HopQ. We first analyzed whether HP0231 influenced the expression of HopQ in different *H. pylori* strains, and observed that the deletion of *hp0231* did not affect the expression of HopQ (Figure 3a). To assess whether HopQ was still functional in the HP0231 KO strain, we performed pull-down assays using recombinant CEACAM1. We observed that HP0231-deficient bacteria were still able to pull down recombinant CEACAM1, whereas *ΔhopQ* strains were unable to do so (Figure 3b). To substantiate these results, we performed binding assays to human gastric epithelial cells. The deletion of *hopQ* led to a significant reduction in *H. pylori* binding to MKN28-CEACAM1 cells. In contrast, no changes in binding to the same cell lines were detected for strains lacking HP0231 (Figure 3c), confirming our previous observations [7]. We corroborated these results using confocal microscopy. We barely observed *H. pylori* binding to MKN28-CEACAM1 cells in the absence of HopQ (Figure 3d), while similar amounts of wild type and Δ*hp0231* bacteria were observed to attach to the gastric epithelial cells. 

Together, our results demonstrate that HP0231 does not influence *H. pylori* binding to gastric epithelial cells.

### 3.3. HopQ-CEACAM1 Mediated CagA Translocation is Dependent on the CL1 Disulfide

**Figure 4:**
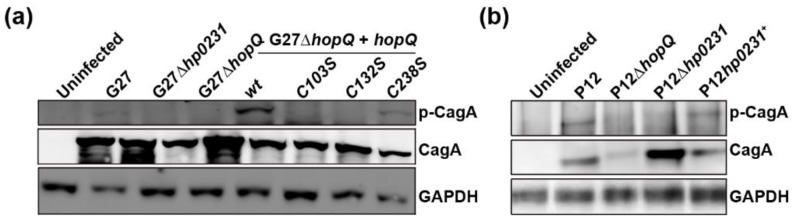
HopQ CL1 mutants are unable to translocate CagA. CagA, p-CagA, and GAPDH protein levels detected by Western blot in lysates of MKN28-CEACAM1-4L cells infected with different G27 (**a**) or P12 (**b**) *H. pylori* strains.

We have previously shown that HopQ-CEACAM1 interaction is essential for CagA injection into host cells [3]. Once injected, CagA is phosphorylated and activates a number of signaling pathways that contribute to malignant transformation of the gastric mucosa. Having observed impaired binding of CL1 mutants to CEACAM1 (Figure 2), we assessed the ability of the different mutant strains to translocate CagA into gastric epithelial cells, by detecting phosphorylated CagA in cell lysates. We found that CL1 mutants were deficient in CagA translocation (Figure 4a,b), as was the ∆*hp0231* mutant (Figure 4a,b), as previously published [7].

## 4. Discussion

Previously we described the importance of the HopQ–CEACAM interaction to *H. pylori* pathogenesis [3]. The co-complex structure between HopQ and CEACAM1 provided molecular details on the interaction and revealed the dependence on disulfide-clasped loops to form H-bonds and hydrophobic interactions with the N-terminal domain of CEACAM1 [5]. In this study, we demonstrate that the disulfide bond between residues C103 and C132, present in cysteine-clasped loop CL1, is required for CEACAM1 engagement by HopQ, whereas the disulfide bonds present in CL2 and 3are not. This is in contrast to what was reported in a recent paper [23], though consistent with our published co-complex structure [5]. As CL3 is located distant from the binding interface, no negative effect upon disruption of its disulfide is expected. However, the disulfide bond from CL2 is part of the HopQ–CEACAM1 binding interface and forms a platform that provides hydrophobic contacts to the interaction. It should be noted that CL2 does not contribute any direct H-bond to the interaction. This can explain why the loss of the CL2 disulfide bond does not affect HopQ-mediated binding, as any resulting conformational change would not directly disrupt H-bonds, and CL2 can still perform its function as a supporting platform. Indeed, the only specific CL2 residues that interact with CEACAM1, Ile240 and Ile242, which form hydrophobic interactions with CEACAM1, are completely absent from the HopQ type II family, an alternative family of HopQ proteins that still maintains CEACAM binding [5]. In contrast, CL1 residue Tyr106 forms a key H-bond with CEACAM1 residue Thr56, and the loss of this H-bond has been shown to significantly disrupt the HopQ–CEACAM1 interaction [5]. Reducing the disulfide bond at the base of CL1 could alter its conformation to a non-functional state and abrogate this H-bond formation. CL1 also provides a scaffold for other structural elements of HopQ, namely the loops CL1-H3 and CL1-H4 (the loops between CL1 and α-helices 3 and 4, respectively). Disruption of these loops would result in a loss of up to 8 H-bonds, and can also help to explain the observed loss of binding. The finding that HopQ–CEACAM5 binding can be disrupted in a similar manner suggests that HopQ depends on similar strategies for binding both CEACAM1 and 5.

With the importance of the disulfide in CL1 to the HopQ–CEACAM interaction revealed, the next question was whether the Dsb-like oxidoreductase HP0231 is the enzyme responsible for the formation of this disulfide. We have previously shown that HP0231 is necessary for *H. pylori* virulence, as HP0231 mutants demonstrated reduced gastric colonization and were unable to translocate CagA into eukaryotic cells [7]. However, our results indicate that this is independent of HopQ, since the lack of HP0231 did not impair HopQ–CEACAM1 interaction, and thus the binding of *H. pylori* to epithelial cells. This finding is in contrast to a recent description of the interaction of HP0231 with HopQ, where HP0231 was thought to directly interact with and influence HopQ binding and subsequent CagA translocation [23]. The discrepancy in strains used or assay formats could explain the contrasting findings we observed, though in our assays we found that ∆*hp0231* mutants in multiple strain backgrounds had no effect on HopQ-mediated binding in several assay settings. In the context of both studies, it could be proposed that HP0231 acts in concert with another Dsb-like protein. Two other Dsb-like proteins were reported in *H. pylori*—HP0595 (HpDsbI), which has been described as impacting HP0231 reoxidation [13], and HP0377, a DsbC-like protein which has been described as depending on HP0231 for redox homeostasis [14]. A possible link between HP0595 and HopQ function was ruled out, since HP0595-deficient bacteria were still able to translocate CagA into host cells [23]. HP0377, like HP0231, is localized in the periplasm, making this enzyme the most likely candidate for direct interaction with HopQ. However, the deletion of HP0377 is not possible, as it is an essential protein for *H. pylori*. Thus, it is difficult to assess its role in HopQ disulfide bond formation.

Preventing host–receptor interaction has been touted as a novel therapeutic approach to infectious disease. One approach that has proven successful in the past is the use of peptides to block pathogen binding. This has been demonstrated in approaches targeting viruses, such as Hepatitis B [24], human cytomegalovirus [25], influenza virus [26] and others [27], and, more recently, bacteria such as *Mycobacterium tuberculosis* [28]. Another approach is the use of antibodies to block the host-pathogen interaction [3], as well as nanobodies that overcome the limitations of traditional monoclonal antibodies and peptides as therapeutics [29,30]. An alternative approach that is prompted by identifying the importance of the CL1 disulfide bond in this study, and also avoids the limitations of other strategies, is to design small molecules that target and reduce the disulfide bond [31,32], thus preventing *H. pylori* binding; an approach that has previously been used to target HIV infection [33,34]. In this context, a previous study showed that binding of the *H. pylori* adhesin BabA to its cellular receptor Le^b^ was impaired by the reduction in a conserved 8-residue cysteine-clasped loop in the adhesins glycan-binding site [8]. The treatment of *H. pylori* cells with the approved redox-active pharmaceutical *N*-acetyl cysteine (NAC) resulted in the loss of BabA-dependent binding to the gastric mucosa and the treatment of mice infected with *H. pylori* with a two-week regime of orally administered NAC resulted in a reduced bacterial colonization and gastric inflammation.

Studying the structural elements of HopQ-CEACAM interaction has allowed us to demonstrate a key and specific role for disulfide bonds in *H. pylori* virulence, but, more importantly, offered a broader view of the larger network of proteins that supports what initially seems a straightforward protein–protein interaction. Although HopQ has been proposed to interact with the T4SS [35], the impaired CagA-translocation observed in our study is a result of the binding defect induced by the mutations inserted. The clear importance of Dsb-like proteins to *H. pylori* infection, whether it be HopQ binding, CagA translocation or others, prompts further work to precisely identify their contributions and further our understanding of their roles.
